# Association of body temperature and mortality in critically ill patients: an observational study using two large databases

**DOI:** 10.1186/s40001-023-01616-3

**Published:** 2024-01-06

**Authors:** Daniel J. Tan, Jiayang Chen, Yirui Zhou, Jaryl Shen Quan Ong, Richmond Jing Xuan Sin, Thach V. Bui, Anokhi Amit Mehta, Mengling Feng, Kay Choong See

**Affiliations:** 1 Institute of Data Science, National University of Singapore, Singapore, Singapore; 2 National University Hospital, Singapore, Singapore; 3 School of Computing, National University of Singapore, Singapore, Singapore; 4 Faculty of Science, National University of Singapore, Singapore, Singapore; 5 Faculty of Engineering, National University of Singapore, Singapore, Singapore; 6 Indian Institute of Technology Bombay, Bombay, India; 7 Saw Swee Hock School of Public Health, National University of Singapore, Singapore, Singapore; 8 Division of Respiratory and Critical Care Medicine, Department of Medicine, National University Hospital, Singapore, Singapore

**Keywords:** Body temperature, Critical care, Electronic health records, Fever, Hypothermia, Therapeutic hypothermia

## Abstract

**Background:**

Body temperature (BT) is routinely measured and can be controlled in critical care settings. BT can impact patient outcome, but the relationship between BT and mortality has not been well-established.

**Methods:**

A retrospective cohort study was conducted based on the MIMIC-IV (*N* = 43,537) and eICU (*N* = 75,184) datasets. The primary outcome and exposure variables were hospital mortality and first 48-h median BT, respectively. Generalized additive models were used to model the associations between exposures and outcomes, while adjusting for patient age, sex, APS-III, SOFA, and Charlson comorbidity scores, temperature gap, as well as ventilation, vasopressor, steroids, and dialysis usage. We conducted subgroup analysis according to ICU setting, diagnoses, and demographics.

**Results:**

Optimal BT was 37 °C for the general ICU and subgroup populations. A 10% increase in the proportion of time that BT was within the 36–38 °C range was associated with reduced hospital mortality risk in both MIMIC-IV (OR 0.91; 95% CI 0.90–0.93) and eICU (OR 0.86; 95% CI 0.85–0.87). On the other hand, a 10% increase in the proportion of time when BT < 36 °C was associated with increased mortality risk in both MIMIC-IV (OR 1.08; 95% CI 1.06–1.10) and eICU (OR 1.18; 95% CI 1.16–1.19). Similarly, a 10% increase in the proportion of time when BT > 38 °C was associated with increased mortality risk in both MIMIC-IV (OR 1.09; 95% CI 1.07–1.12) and eICU (OR 1.09; 95% CI 1.08–1.11). All patient subgroups tested consistently showed an optimal temperature within the 36–38 °C range.

**Conclusions:**

A BT of 37 °C is associated with the lowest mortality risk among ICU patients. Further studies to explore the causal relationship between the optimal BT and mortality should be conducted and may help with establishing guidelines for active BT management in critical care settings.

**Supplementary Information:**

The online version contains supplementary material available at 10.1186/s40001-023-01616-3.

## Introduction

Body temperature (BT) is one of the most important vital signs of critically ill patients [1]. It is commonly used as a biomarker for detecting infection and its fluctuations are frequently observed among critically ill patients [2]. BT is additionally used as a therapeutic target for management in the critical care setting, and it can be adjusted with varying degrees of precision using available technologies [3, 4]. More precise physical methods of cooling include the use of cooling blanket systems and infusion of cooled intravenous fluids, whereas less precise pharmacological methods of cooling include the use of antipyretic medications like paracetamol and non-steroidal anti-inflammatory drugs [5, 6]. In addition, controlled warming devices exist such as counter-current heat exchangers, heat insulators, and warming mattresses [7].

Numerous studies have examined the impact of BT on clinical outcomes in intensive care unit (ICU) patients with a range of conditions [8]. Both hypothermia and fever are associated with worse mortality outcomes among general ICU patients [9, 10], with an incremental mortality increase for every 1 °C deviation from normothermia [8, 9, 11]. However, the definitions of hypothermia and fever are variable, and the optimal BT ranges are unclear [12–14]. For example, in one study fever was defined as BT greater than 37.5 °C and hypothermia as a BT lower than 36.5 °C [12], while in other studies the temperature cut-offs are different [14]. Furthermore, the reported influence of BT on mortality among critically ill patients has been mixed [1, 3, 11, 15–18]. Among ICU patients with brain pathologies, no difference in survival has been observed with or without temperature management in one study [4], but fever and hypothermia seemed to have worse mortality outcomes in other studies [12, 19, 20]. Among septic patients, hypothermia was associated with increased mortality, while fever was not [17, 21]. Although ICU severity scoring systems such as APACHE do take BT into account, it remains uncertain if the temperature ranges defined by the system apply to all subgroups, especially since the calibration and predictive performance of the score is known to vary widely across patient disease and demographic subgroups [22, 23].

Given the uncertainty around the definitions of hypothermia and fever, as well as the variability in reported associations of BT with mortality, our study aims to derive and validate an optimal BT associated with minimum mortality for critically ill patients including various subgroups. We hypothesize that a U-shaped distribution exists between BT and mortality, with the extremes of low and high temperature being associated with increased mortality. In addition, we aim to investigate if the difference between the maximum and minimum BT measurement (i.e., the temperature gap) is also associated with increased mortality. The study results can help define hypothermia and fever and guide targeted temperature management for ICU patients.

## Methods

### Data source and cohort selection

Our study was designed following the Strengthening the Reporting of Observational Studies in Epidemiology (STROBE) reporting guidelines [24]. It was conducted using two datasets, the Medical Information Mart for Intensive Care IV (MIMIC-IV) and eICU Collaborative Research Database (eICU). MIMIC-IV captures over a decade of patient ICU stays (from 2008 to 2019) at the Beth Israel Deaconess Medical Center and contains information for over 60,000 total ICU patients [25]. On the other hand, eICU contains data for over 200,000 ICU admissions from 2014 to 2015 monitored by eICU-affiliated programs across the United States [26]. Since the eICU dataset comprises data from many distinct participant hospitals with customized workflows and clinical documentation processes, data reliability and completion differ at the hospital level. To address this, we only included data from hospitals with at least 95% completion of fluid input and output documentation within the eICU database. For both datasets, we considered only each patient’s first ICU stay and only included adult patients (≥ 18 years of age) with at least five different BT readings within their first 48 h of ICU admission.

### Variables and pre-preprocessing

For each patient, we collected dynamic BT data within 48 h of their ICU admission, derived median BTs, and computed the temperature gap (maximum–minimum BT). To minimize inclusion of questionable temperature readings we set a plausible BT range to be 30–45 °C, excluding measurements outside this range. BTs were assumed to be constant in between recorded measurements. Our main and secondary outcome measures were hospital mortality and ICU mortality, respectively. We also collected demographic, diagnosis, drug usage, and procedure information. Diagnoses were collected using ICD-9 and ICD-10 codes; specifically, we considered atrial fibrillation, cancer, cardiac arrest, chronic kidney disease, chronic liver disease, chronic obstructive pulmonary disease, diabetes, hypertension, ischemic heart disease, and traumatic brain injury (TBI). For cardiac arrest, sepsis, stroke, and TBI, we included only patients who had these conditions coded as a primary diagnosis. Other variables collected or calculated included sex, age, dialysis, steroid use (hydrocortisone, prednisone, prednisolone, cortisone, dexamethasone), vasopressor use, invasive ventilation within the first 48 h of admission, APS-III score, SOFA score, Charlson Comorbidity Score, ICU type, and emergency admission. SOFA and Charlson Comorbidity scores were directly provided in the MIMIC database. To derive SOFA and Charlson scores for eICU, we used the code provided by Sarkar et al*.* [27] and Chandra et al*.* [28], respectively.

### Statistical analysis

Generalized Additive Models (GAM) were utilized to analyze the association between our exposure variables and the target outcomes. All GAM models were adjusted for the following covariates: age at admission, sex, APS-III score, SOFA score, Charlson comorbidity score, temperature gap, as well as steroid, ventilation, vasopressor, and dialysis usage within the first 48 h of admission. For each of these covariates as well as for the patient subgroupings, we measured their association with hospital mortality using a Wilcoxon rank sum test and Chi-squared test for continuous and categorical variables, respectively.

We used a priori clinical evidence in addition to a stepwise regression modeling to aid in the selection of these covariates. Age and sex have been repeatedly demonstrated to be associated with BT: BT decreases with age [29–31] and is higher in women than men [32, 33]. Vasopressin is known to produce antipyretic effects during fever [34, 35], while steroids may have varying effects on BT depending on the type of steroid and the patient’s condition [36, 37]. Dialysis is also included as it may influence BT, since blood that is returned to the patient is in thermal equilibrium with the dialysate [38]. Moreover, we adjusted for ventilation use due to its association with mortality as well as temperature gap to account for swings in the patients’ BT.

We statistically validated the importance of the chosen covariates through stepwise forward and backward variable selection procedures [39] using the AIC criterion. This was carried out using MIMIC-IV data with median BT and hospital mortality as the exposure and outcome variables of interest, respectively (Additional file 6: Table S1).

For eICU, given potential heterogeneity across different hospitals, statistical models included hospital ID as a random slope term. We also attempted to determine an appropriate temperature gap for analysis. Should there be no inflection point when temperature gap is plotted against mortality, we planned to use a gap of ± 1 °C, as randomized control trials of targeted temperature management were generally able to control BT within 2 °C around a target temperature [40, 41].

Three types of GAMs were fitted, each with a different exposure variable and with hospital mortality as the target outcome; each type of model was fitted on both the MIMIC-IV and eICU datasets separately. The first GAM model incorporated median BT as the main exposure variable and modeled the variable using cubic splines. The second GAM incorporated temperature gap as the exposure variable and modeled the variable with cubic splines. The number of knots chosen for the cubic spline terms were determined by splitting the data into 80% training and 20% testing sets, then fitting a GAM using 5–15 knots for spline terms and selecting the number which maximized the area under the ROC on the training set. The third GAM incorporated the proportion of BTs within a specified BT range as the exposure variable and modeled this term linearly. These BT ranges used were prespecified as the threshold at which temperature gap was associated with increased mortality, or ± 1 °C of the optimal BT, if there was no clear threshold found for temperature gap. Odds ratios of hospital mortality and ICU mortality were calculated for every 10% increase in time within the specified optimal BT ranges for both MIMIC-IV and eICU datasets.

In our subgroup analyses, we repeated the same analyses to identify optimal BTs as well as to calculate the odds ratios associated with every 10% increase in time spent within the specified optimal BT range. Subgroups included in our analyses are as follows: cardiac ICU patients, medical ICU patients, neurosurgical ICU patients, surgical ICU patients, atrial fibrillation, cancer, cardiac arrest, chronic kidney disease, chronic liver disease, congestive heart failure, chronic obstructive pulmonary disease, diabetes, hypertension, ischemic heart disease, non-septic cardiac arrest, sepsis, septic cardiac arrest, stroke, TBI, Asian, Black, Hispanic, White patients, patients aged ≥ 75 years, emergency admission, and acetaminophen usage.

### Analytical software

Statistical analyses were carried out using Python (version 3.10) and the packages Scikit-learn (version 1.2.2), PyGam (version 0.8.0), and NumPy (version 1.24.3).

### Code availability

https://github.com/nus-mornin-lab/temperature_paper_2023.

## Results

We included 43,537 and 75,184 unique ICU admissions from the MIMIC-IV and eICU datasets, respectively. Hospital and ICU mortality rates were 9.3% and 6.2% for MIMIC-IV and 9.2% and 5% for eICU. Patient demographics, median APS-III, SOFA, and Charlson Comorbidity Index were similar across both datasets. For MIMIC, rates of emergency admission (53.3%), invasive ventilation (28.2%), and steroid usage (16.2%) were higher than that of eICU (39.1%, 24.5%, and 9.3% for emergency admission, invasive ventilation, and steroid usage respectively). However, dialysis usage was similar (3.1% and 3.3% for MIMIC and eICU) and vasopressor usage was higher in eICU (10.1%) vs MIMIC-IV (4%) (Table 1).

**Table 1:** Patient cohort characteristics for MIMIC-IV and eICU datasets

Patient characteristics	MIMIC-IV (*N* = 43537)	eICU (*N* = 75184)	MIMIC-IV Hosp. Mortality association P-Value	eICU Hosp. Mortality association *P*-Value
Temperature (°C)	36.9 (36.6–37.2)	37.1 (36.6–37.6)	< 0.001	< 0.001
Temperature gap (°C)	1.1 (0.7–1.7)	1.1 (0.7–1.6)	< 0.001	< 0.001
Hospital death	4069 (9.3%)	6931 (9.2%)	NA	NA
ICU death	2678 (6.2%)	3784 (5%)	NA	NA
Sex (Male)	24368 (56%)	40402 (53.7%)	0.002	0.173
Age (years)	66.8 (54.8–78.1)	65 (53–76)	< 0.001	< 0.001
APS-III	39 (29–54)	40 (29–52)	< 0.001	< 0.001
SOFA	3 (1–5)	2.5 (1–4.5)	< 0.001	< 0.001
Charlson score	5 (3–7)	4 (2–6)	< 0.001	< 0.001
Emergency	23212 (53.3%)	29384 (39.1%)	< 0.001	0.931
Ventilation	12273 (28.2%)	18418 (24.5%)	< 0.001	< 0.001
Vasopressor usage	1748 (4%)	7582 (10.1%)	< 0.001	< 0.001
Dialysis	1354 (3.1%)	2448 (3.3%)	< 0.001	< 0.001
Steroid usage	7033 (16.2%)	7026 (9.3%)	< 0.001	0.61
CVICU + CCU	7560 (17.4%)	7773 (10.3%)	< 0.001	0.299
MICU	8255 (19%)	6623 (8.8%)	< 0.001	< 0.001
Neuro SICU	1236 (2.8%)	9632 (12.8%)	< 0.001	0.011
SICU	5695 (13.1%)	4953 (6.6%)	0.816	< 0.001
Atrial fibrillation	5950 (13.7%)	6174 (8.2%)	< 0.001	< 0.001
Cancer	5421 (12.5%)	5326 (7.1%)	< 0.001	< 0.001
Cardiac arrest	22 (0.1%)	1480 (2%)	< 0.001	< 0.001
Chronic kidney disease	7485 (17.2%)	4771 (6.3%)	< 0.001	< 0.001
Chronic liver disease	4174 (9.6%)	809 (1.1%)	< 0.001	< 0.001
Congestive heart failure	9574 (22%)	5965 (7.9%)	< 0.001	< 0.001
Chronic obstructive pulmonary disorder	9672 (22.2%)	6378 (8.5%)	< 0.001	0.06
Diabetes	7208 (16.6%)	7140 (9.5%)	0.751	0.031
Hypertension	23347 (53.6%)	9500 (12.6%)	0.192	< 0.001
Ischemic heart disease	13780 (31.7%)	5642 (7.5%)	0.014	0.258
Non-septic cardiac arrest	276 (0.6%)	1457 (1.9%)	< 0.001	< 0.001
Sepsis	20369 (46.8%)	3910 (5.2%)	< 0.001	< 0.001
Septic cardiac arrest	594 (1.4%)	23 (0%)	< 0.001	< 0.001
Stroke	2959 (6.8%)	3153 (4.2%)	< 0.001	< 0.001
Traumatic brain injury	1808 (4.2%)	1202 (1.6%)	< 0.001	0.24

Data are presented as median (IQR) or *N* (%)

**Fig. 1:**
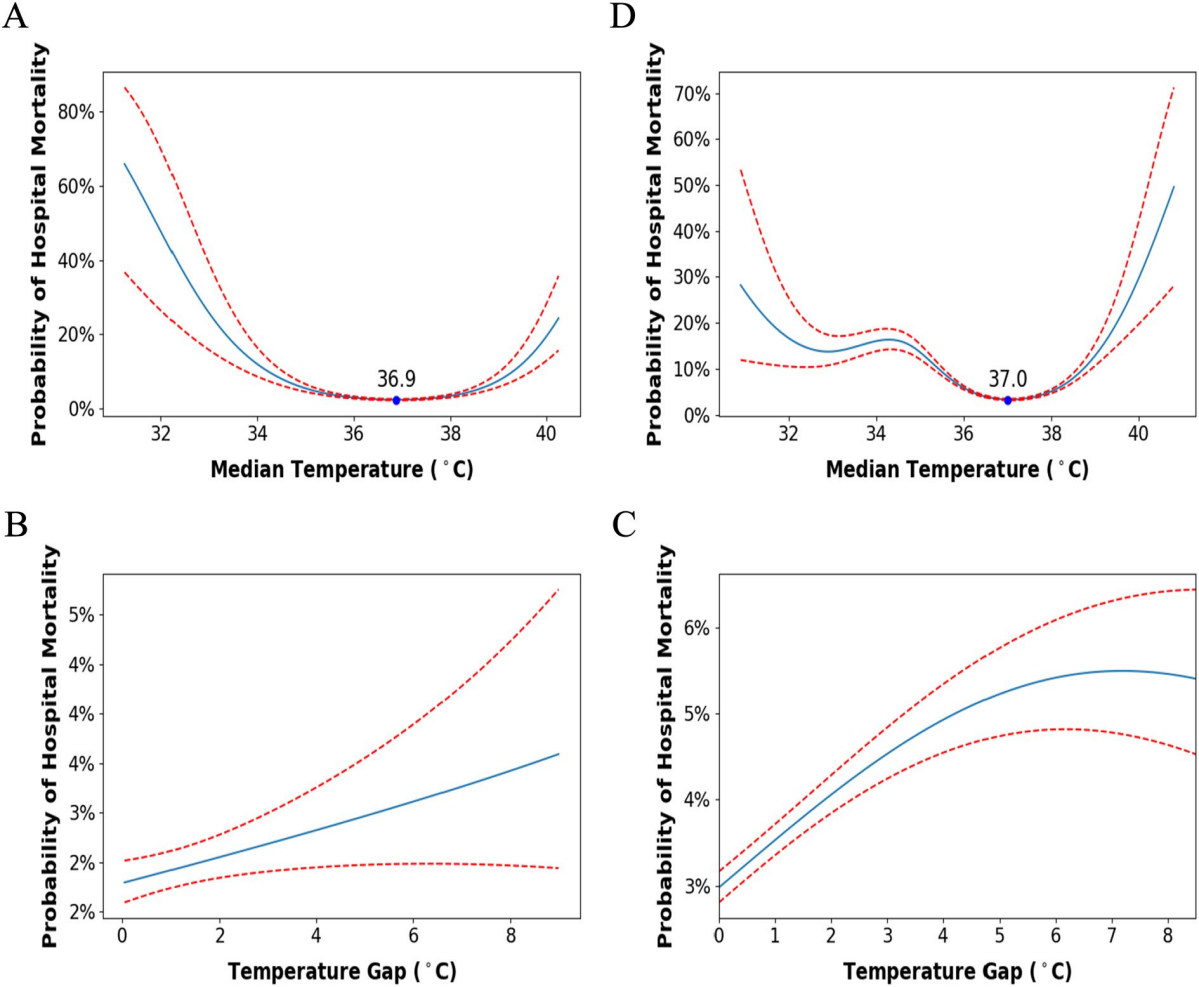
Probability of hospital mortality vs median BT for **A** MIMIC-IV and **B** eICU. Probability hospital mortality vs temperature gap in the first 48 h of ICU stay for **C** MIMIC-IV and **D** eICU. All models are adjusted for age, sex, APS-III score, Charlson Comorbidity Score, SOFA score, ventilation usage, vasopressor usage, steroids usage, dialysis usage. **A** and **B** are additionally adjusted for temperature gap, while **B** and **C** are adjusted for median temperature

We found a U-shaped relationship between median BT and odds of hospital mortality in our ICU patient cohorts (Fig. 1A, B). The optimal BTs found (as given by the x-axis of minimum point of the U-shaped curve) were 36.8 and 37.0 °C for MIMIC-IV and eICU, respectively. We considered the overall optimal BT to be 37 °C. On the other hand, temperature gap was found to have a positive linear relationship with hospital mortality in both MIMIC-IV (Fig. 1C) and eICU cohorts (Fig. 1D), with no threshold found. We therefore specified a BT range of 37 ± 1 °C (i.e., 36–38 °C) to analyze the association of mortality with proportion of BTs within the specified BT range.

**Fig. 2:**
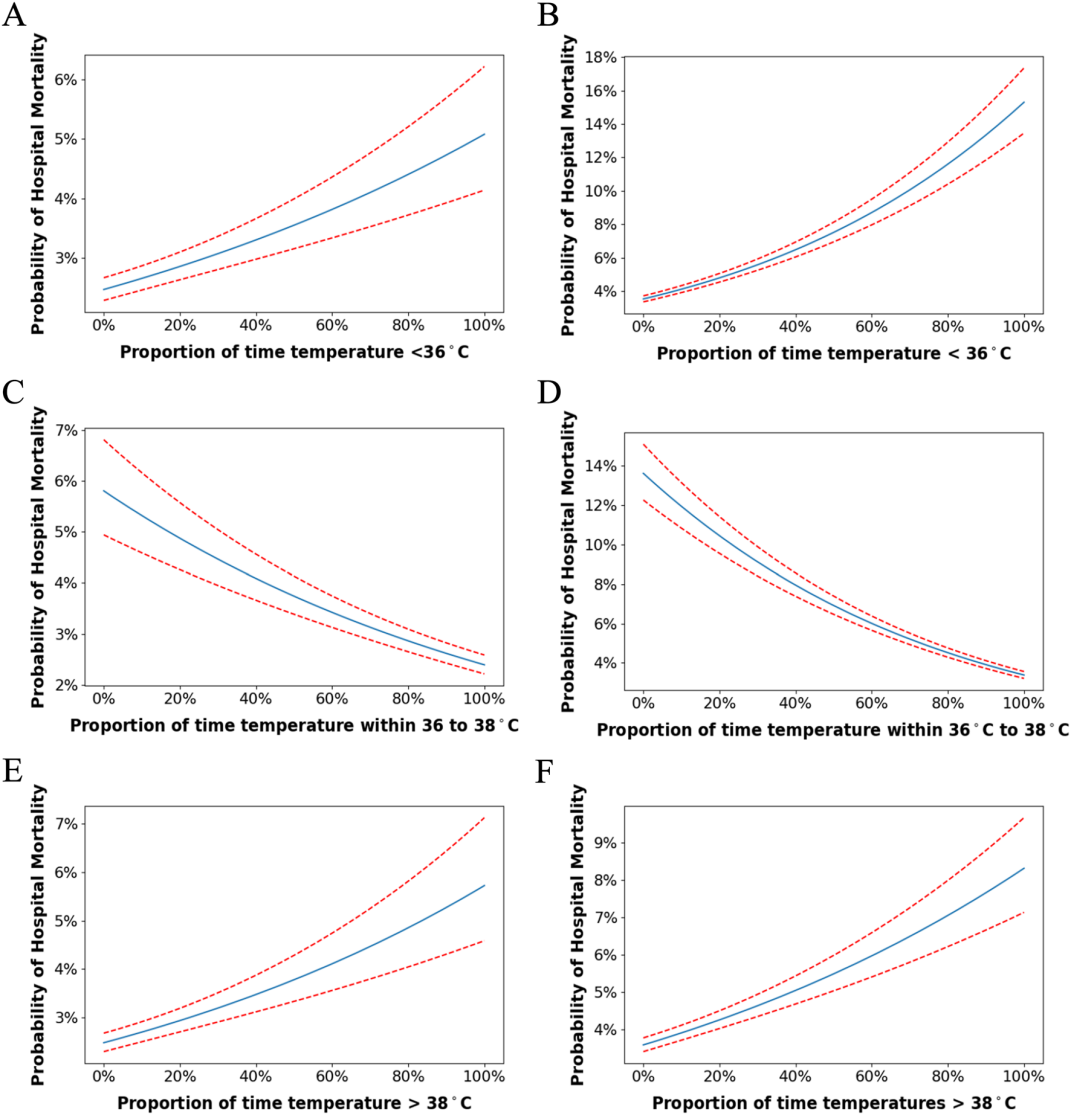
Probability of hospital mortality vs proportion of time BT < 36 °C for **A** MIMIC and **B** eICU, BT between 36 and 38 °C for **C** MIMIC-IV and **D** eICU, BT > 38 °C for **E** MIMIC-IV and **F** eICU. Adjusted for age, sex, APS-III score, Charlson Comorbidity Score, SOFA score, ventilation usage, vasopressor usage, steroids usage, dialysis usage, and temperature gap

Increased time spent with a BT < 36 °C was associated with increased risk of hospital mortality (Fig. 2A, B). A 10% increase in time spent with a BT < 36 °C was associated with 8–18% increased odds of hospital mortality: adjusted odds ratio (OR) of 1.08 (95% CI 1.06–1.10) in the MIMIC-IV cohort and OR of 1.18 (95% CI 1.16–1.19) in the eICU cohort (Table 2). Increased time spent with a BT within 36–38 °C was associated with decreased risk of hospital mortality (Fig. 2C, D). A 10% increase in time spent with a BT within 36–38 °C was associated with 9–14% decreased odds of hospital mortality: adjusted OR of 0.91 (95% CI 0.90–0.93) in the MIMIC-IV cohort and odds ratio of 0.86 (95% CI 0.85–0.87) in the eICU cohort (Table 2). Increased time spent with a BT > 38 °C was associated with increased risk of hospital mortality (Fig. 2A, B). A 10% increase in time spent with a BT > 38 °C was associated 9% increased odds of hospital mortality: adjusted OR of 1.09 (95% CI 1.07–1.12) in the MIMIC-IV cohort and OR of 1.09 (95% CI 1.08–1.11) in the eICU cohort (Table 2). Unadjusted ORs are presented in Additional file 7: Table S2.

**Table 2:** Adjusted odds ratio of mortality at different temperature ranges

Temperature	MIMIC-IV		eICU	
	Hospital mortality	ICU mortality	Hospital mortality	ICU mortality
< 36 °C	1.08 (1.06–1.1)	1.11 (1.08–1.14)	1.18 (1.16–1.19)	1.2 (1.18–1.22)
36–38 °C	0.91 (0.9–0.93)	0.89 (0.87–0.91)	0.86 (0.85–0.87)	0.84 (0.83–0.85)
> 38 °C	1.09 (1.07–1.12)	1.1 (1.07–1.13)	1.09 (1.08–1.11)	1.1 (1.08–1.12)

**Fig. 3:**
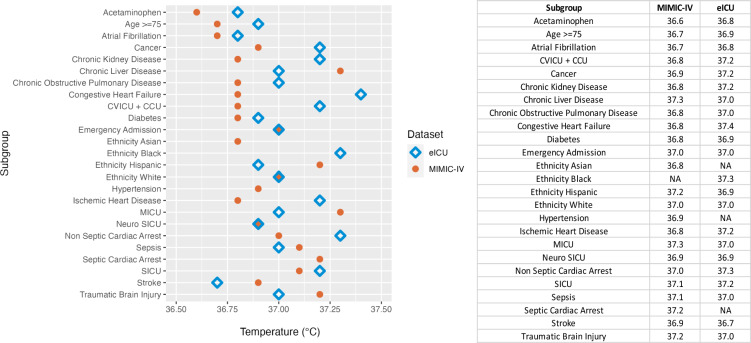
Optimal BT for patient subgroups. *NA measurements indicate that a U-shaped relationship was not found for the subgroup

Across most of the subgroups tested, we also found U-shaped relationships between median BT and odds of hospital mortality (Additional file 1: Figure S1, Additional file 2: Figure S2). All optimal BTs found across the subgroups fell within the 36–38 °C range with slight variations (Fig. 3). ORs of hospital mortality calculated for a 10% increase in time spent within a BT of 36–38 °C for each subgroup ranged from 0.78 to 0.96 (Additional file 3: Figure S3, Additional file 4: Figure S4).

Adjusted odds ratio of hospital and ICU mortality for every 10% increase in total time spent at each temperature range in the first 48 h of ICU stay. ORs are adjusted for age, sex, APS-III score, Charlson Comorbidity Score, SOFA score, ventilation usage, vasopressor usage, steroids usage, dialysis usage, and temperature gap. Data presented as OR (95% CI) (Additional file 5: Figure S5).

## Discussion

In our parallel analysis of two large ICU databases with a total of > 118,000 ICU patients, we determined 37.0 °C to be the BT associated with optimal survival for critically ill patients. Increased time spent outside of the 36–38 °C BT range was independently associated with increased odds of mortality. We also found that minimizing the BT gap (difference of maximum and minimum BT) in the ICU was associated with lower mortality, indicating that BT variability in the ICU was associated with harm. These findings were consistent across the general ICU population and for all subgroups tested.

In clinical publications involving “febrile” patients, the temperature used to define fever was often absent [14]. When a temperature threshold for fever was specified, it has ranged from a low of 37.2 °C to a high of 38.3 °C [14, 42]. To help clarify uncertainty over the definition of fever, based on our results, we propose that fever be defined as BT > 38 °C, normal BT as 36–38 °C, and hypothermia as BT < 36 °C. As we have demonstrated in our results, maintaining patient BTs within the 36–38 °C range confers a survival benefit independent of swings in patient BT.

The detrimental effects of extremes of BT can be explained through various possible physiological mechanisms [43]. Fever involves a rise of the body's core temperature beyond the confines of the hypothalamic set-point temperature and is common among critically ill patients [1]. In extreme cases, fever may contribute to complications such as cardiac arrhythmias, tachycardia, increased oxygen demand, convulsions, and brain damage [18]. The impact of hypothermia on the other hand is less well-understood, though studies suggest that it can undermine immune function by impairing neutrophil and macrophage function [44, 45], induce insulin resistance and hyperglycemia [45], as well as stimulate cold diuresis with risk of hypovolemia [44].

The physiological mechanisms through which extreme BT can impact a patient’s condition are known to vary across different conditions. For example, among patients with coronary heart disease, cold-induced shivering and increased catecholamine release resulting from even mild hypothermia predisposes to myocardial ischemia. This results from the decreased coronary blood flow and increased myocardial oxygenation needs caused by vasoconstriction and increased heart, respiratory, and metabolic rate [46]. Fever, on the other hand, has been linked to infarct expansion [47] and decreased left ventricular function [48].

Among patients with severe sepsis, hypothermia has been linked to increased mortality and organ failure [2]. However, the exact effects of fever on sepsis patients continues to be a subject of contention [49]. There is some evidence to show that antipyretic treatment in sepsis patients leads to worse outcomes [21]. It has also been suggested that in patients with infection, mounting a febrile response can have protective effects via the slowing of micro-organism growth [50] and enhancement of the host immune system [21]. Supporting this, a study by Young and colleagues found a temperature of 39–39.4 °C to be associated with the lowest mortality risk for sepsis patients [51]. On the other hand, fever is known to increase metabolic demand and oxygen consumption of different organs, notably the brain and heart, which can exacerbate the septic patient’s condition [18, 19]. In a mouse model of bacterial pneumonia, fever was shown to decrease survival despite accelerating the elimination of pathogens and enhancing innate host defense. The authors found that fever was associated with increased vascular pulmonary injury, enhanced accumulation of neutrophils, and increased levels of cytokines in bronchoalveolar lavage fluid [52]. Our current findings support the maintenance of normothermia in these patients, with an optimal temperature found to be around 37 °C. However, it should be noted that the decrease in the odds of mortality for every 10% of time spent within the range of normothermia in this group of patients was pronounceably smaller compared to that of other subgroups. Ultimately, the effect of fever in patients with infection is likely determined by a balance between the advantages conferred to host immune response and the adverse metabolic/inflammatory effects of fever [49].

Cardiac arrest patients suffer from high mortality rates due to post-cardiac arrest shock and brain injury, which is known to be exacerbated by fever [53]. Similarly, among patients with neurological pathologies or insults (e.g., stroke or brain injury), fever has been linked to increased length of ICU stay [54] and mortality [12, 19, 20] likely attributable to the aggravation of cerebral metabolic distress. In recent decades, TH has become a popular post-cardiac arrest treatment option, after some studies have shown it to improve neurological recovery in cardiac arrest patients [55]. It has also been proposed for TBI and stroke patients, with the belief that it may reduce damage from excitotoxins, inflammation, free radicals, and necrosis leading to increased neuronal survival [56]. However, more recent randomized trials failed to show any improved functional outcome when compared with strict normothermia in cardiac arrest [57, 58], stroke, and TBI patients [59–61]. Conversely, TH has been linked to increased risk for complications and adverse effects such as pneumonia, hyperglycemia, and cardiac arrhythmias [59]. These trials concur with our results showing an optimal BT of around 37 °C for cardiac arrest, TBI, and stroke patients, and a decrease in odds of mortality in all these subgroups the longer BT is kept within 36–38 °C. An alternative and newer form of TH, selective brain cooling, may avoid the complications associated with systemic, ‘whole-body’ cooling while providing neuroprotective effects [59, 61].

Our study has several limitations. First, given the observational study design, no causal inference between BT and mortality can be made. So, while we showed that BT may serve as a useful predictive biomarker, it should not be used as a therapeutic target without further prospective randomized trials. Second, our study was limited by the accuracy and integrity of the EHR datasets used, though the consistency of results between the two datasets suggest that the results are reliable. Third, we could not consider the site or method of BT temperature readings, as this information was not present in the eICU and incomplete in the MIMIC databases. In the ICU, BT can be measured using intravascular, bladder, esophageal, or rectal probes, or with infrared tympanic membrane and temporal artery thermometers [18]. Small variations may exist among these methods, and some measurements (e.g., oral and axillary) are considered less accurate than others [62]. Fourth, the MIMIC and eICU cohorts represented only ICUs in the US, and further studies from ICUs outside the US will be required to validate our findings.

Our findings should encourage further research in active BT management within 36–38 °C in the ICU to optimize patient outcome. Targeted BT trials should test maintaining an optimal BT within as tight a range as possible. In addition, the existence of an optimal BT range supports the further development of closed loop temperature management devices to achieve more precise temperature control in critically ill patients. On the other hand, our results do not back the use of therapeutic hypothermia (TH), a protocol that has been proposed for post-cardiac arrest, TBI, and stroke patients [55]. Our results concur with several studies that have shown little benefit from TH and strengthen the case for a gradual shift from that strategy [58, 63].

## Interpretation

We found that a BT of 37 °C was associated with optimal outcomes for the critically ill patient population and this result was consistent across various subgroups. Future trials of temperature management could aim to target a BT of 36–38 °C, while minimizing BT variability.

### Supplementary Information

**Additional file 1: Figure S1.** Probability of hospital mortality vs median BT for patient subgroups for MIMIC-IV.

**Additional file 2: Figure S2.** Probability of hospital mortality vs median BT for patient subgroups for eICU.

**Additional file 3: Figure S3.** Adjusted odds ratio of hospital mortality for every 10% increase in time when BT was between 36 °C and 38 °C within first the 48 h of patient’s ICU stay for each patient subgroup.

**Additional file 4: Figure S4.** Adjusted odds ratio of ICU mortality for every 10% increase in time when BT was between 36 °C and 38 °C within first the 48 h of patient’s ICU stay for each patient subgroup.

**Additional file 5: Figure S5.** Average percent of time spent outside 36–36 °C per subgroup.

**Additional file 6: Table S1.** Results of (A) forward and (B) backwards stepwise regression using MIMIC-IV data with median BT as the main exposure and hospital mortality as the outcome.

**Additional file 7: Table S2.** Crude odds ratio of mortality at different temperature ranges.

## Data Availability

Codes used in analysis can be found at the following link: https://github.com/nus-mornin-lab/temperature_paper_2023. Data can be accessed through the following links: https://physionet.org/content/mimiciv/, https://eicu-crd.mit.edu/.

## Abbreviations

APS-III: Acute physiology score III
BT: Body temperature
CCU: Cardiac care unit
CHF: Congestive heart failure
CI: Confidence interval
CKD: Chronic kidney disease
COPD: Chronic obstructive pulmonary disorder
CVICU: Cardiovascular intensive care unit
eICU: EICU Collaborative Research Database
GAM: Generalized additive model
ICD: Ischemic heart disease
ICU: Intensive care unit
MIMIC-IV: Medical information mart for intensive care IV
OR: Odds ratio
SOFA: Sequential organ failure score
TBI: Traumatic brain injury
TH: Therapeutic hypothermia
