# Supplementary figures and images for: Association of body temperature and mortality in critically ill patients: an observational study using two large databases
Source: Eur J Med Res. 2024 Jan 6;29:33. doi: 10.1186/s40001-023-01616-3 (PMC10770998; doi:10.1186/s40001-023-01616-3)

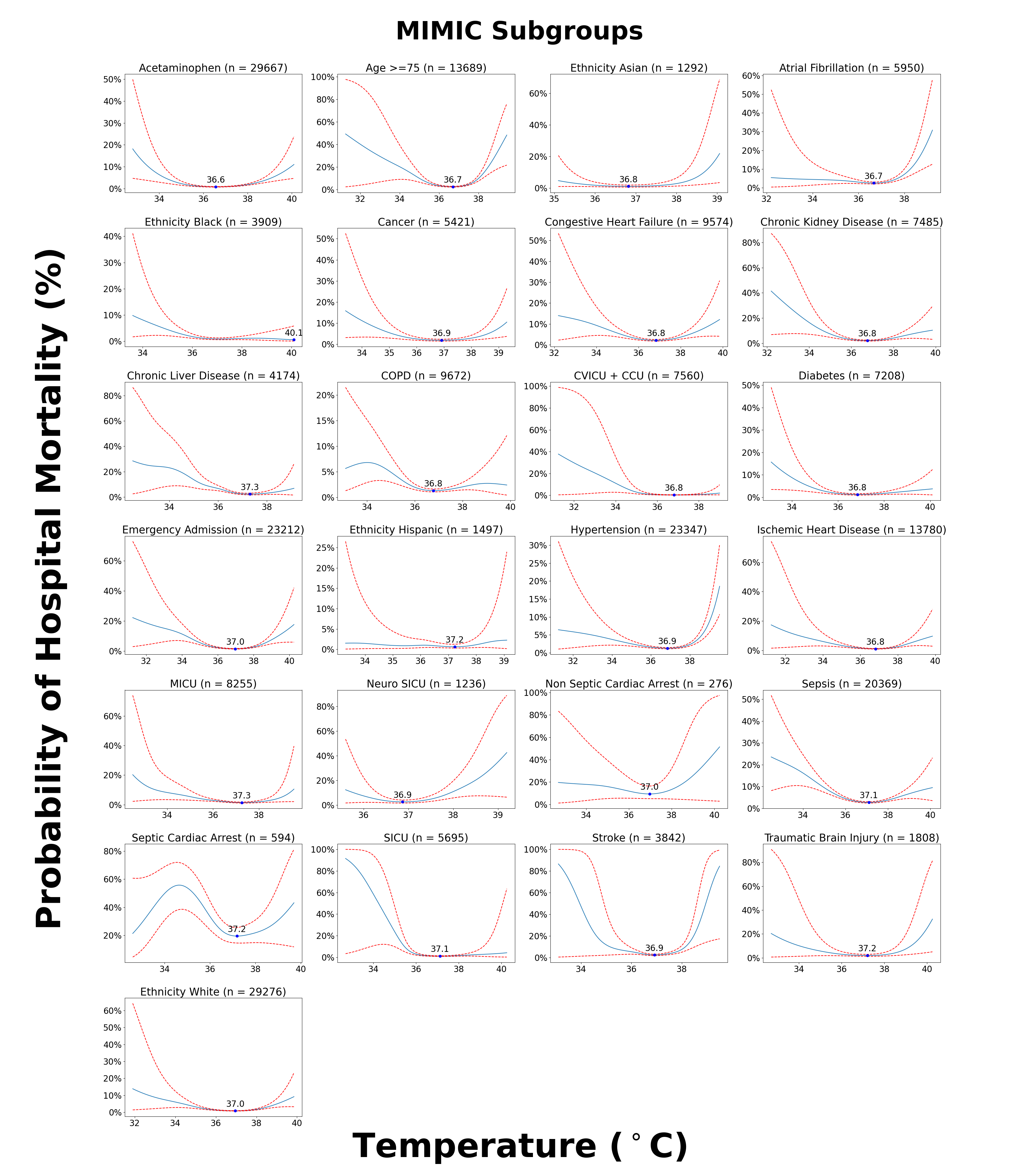

Supplement: Supplementary file 1 — Additional file 1: Figure S1. Probability of hospital mortality vs median BT for patient subgroups for MIMIC-IV. [file 40001_2023_1616_MOESM1_ESM.jpg]

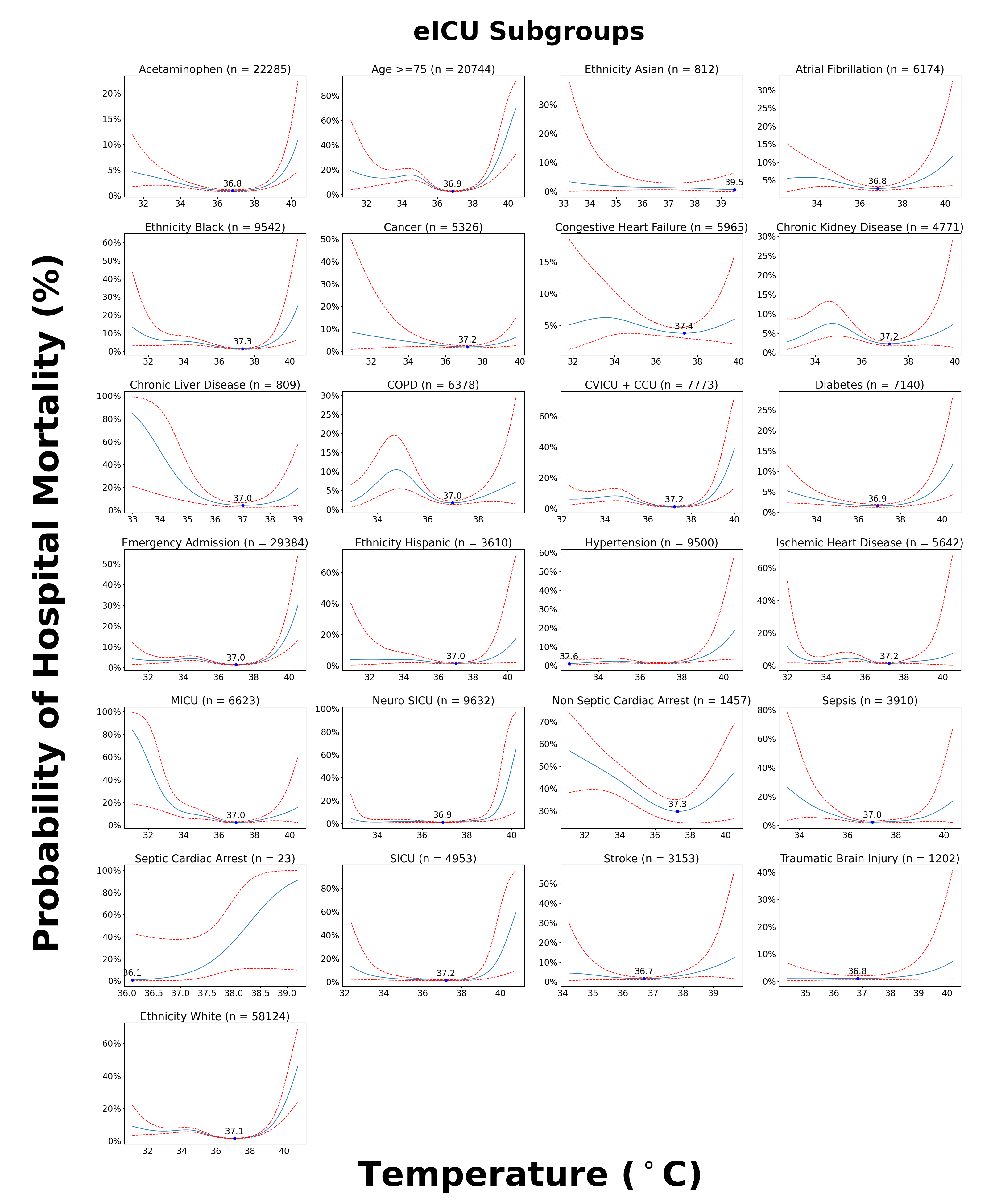

Supplement: Supplementary file 2 — Additional file 2: Figure S2. Probability of hospital mortality vs median BT for patient subgroups for eICU. [file 40001_2023_1616_MOESM2_ESM.jpg]

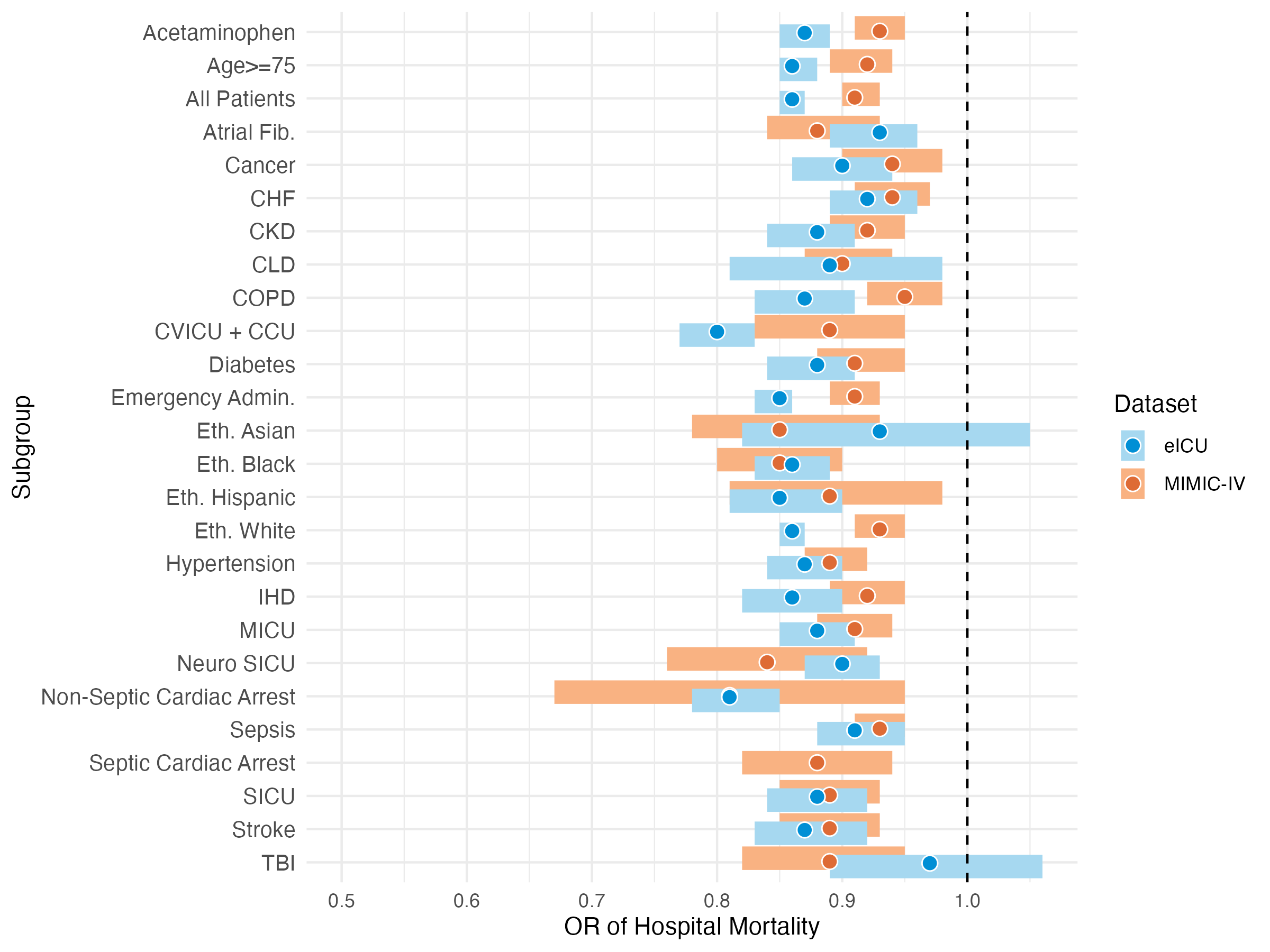

Supplement: Supplementary file 3 — Additional file 3: Figure S3. Adjusted odds ratio of hospital mortality for every 10% increase in time when BT was between 36 °C and 38 °C within first the 48 h of patient’s ICU stay for each patient subgroup. [file 40001_2023_1616_MOESM3_ESM.jpg]

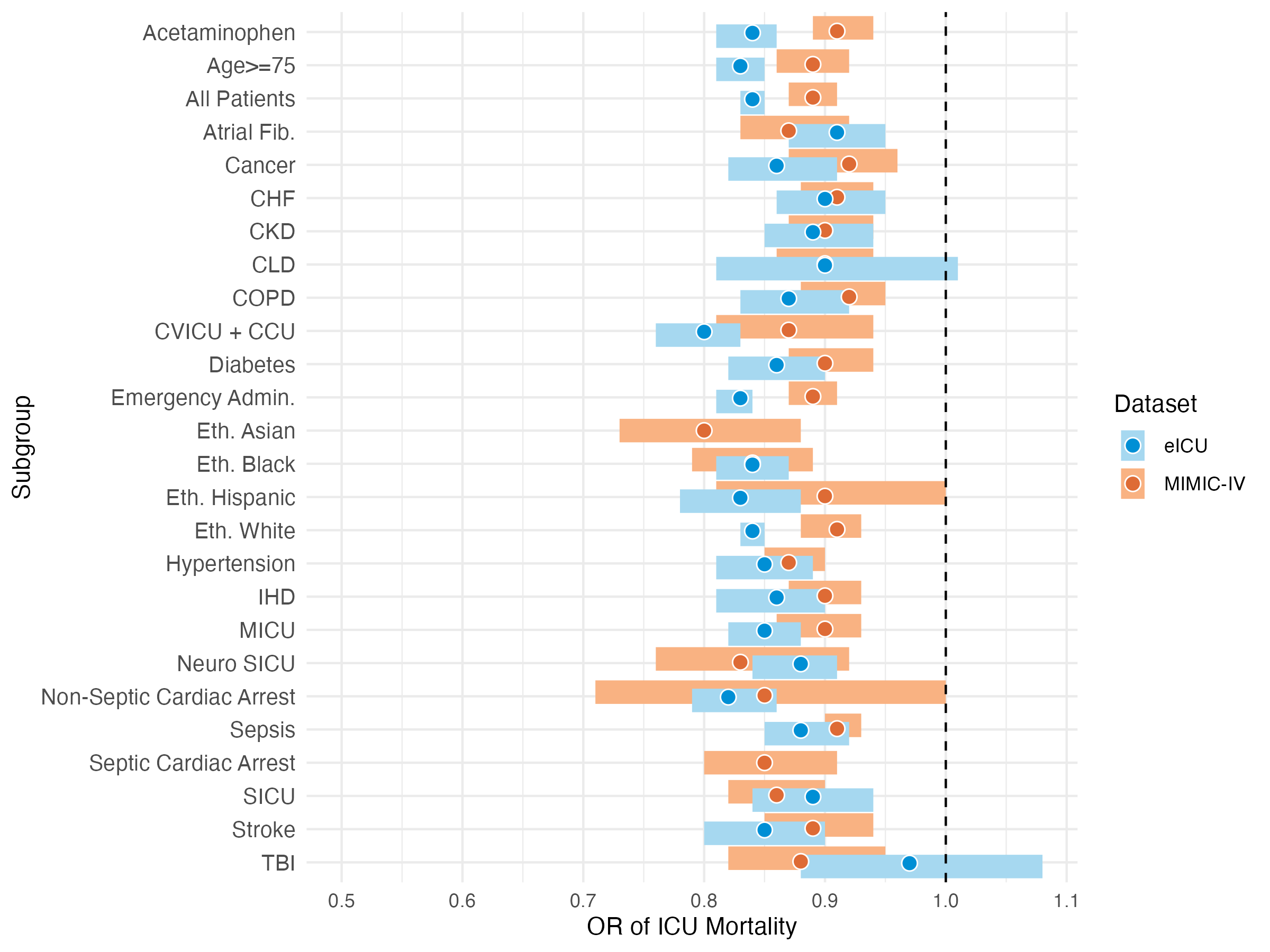

Supplement: Supplementary file 4 — Additional file 4: Figure S4. Adjusted odds ratio of ICU mortality for every 10% increase in time when BT was between 36 °C and 38 °C within first the 48 h of patient’s ICU stay for each patient subgroup. [file 40001_2023_1616_MOESM4_ESM.jpg]

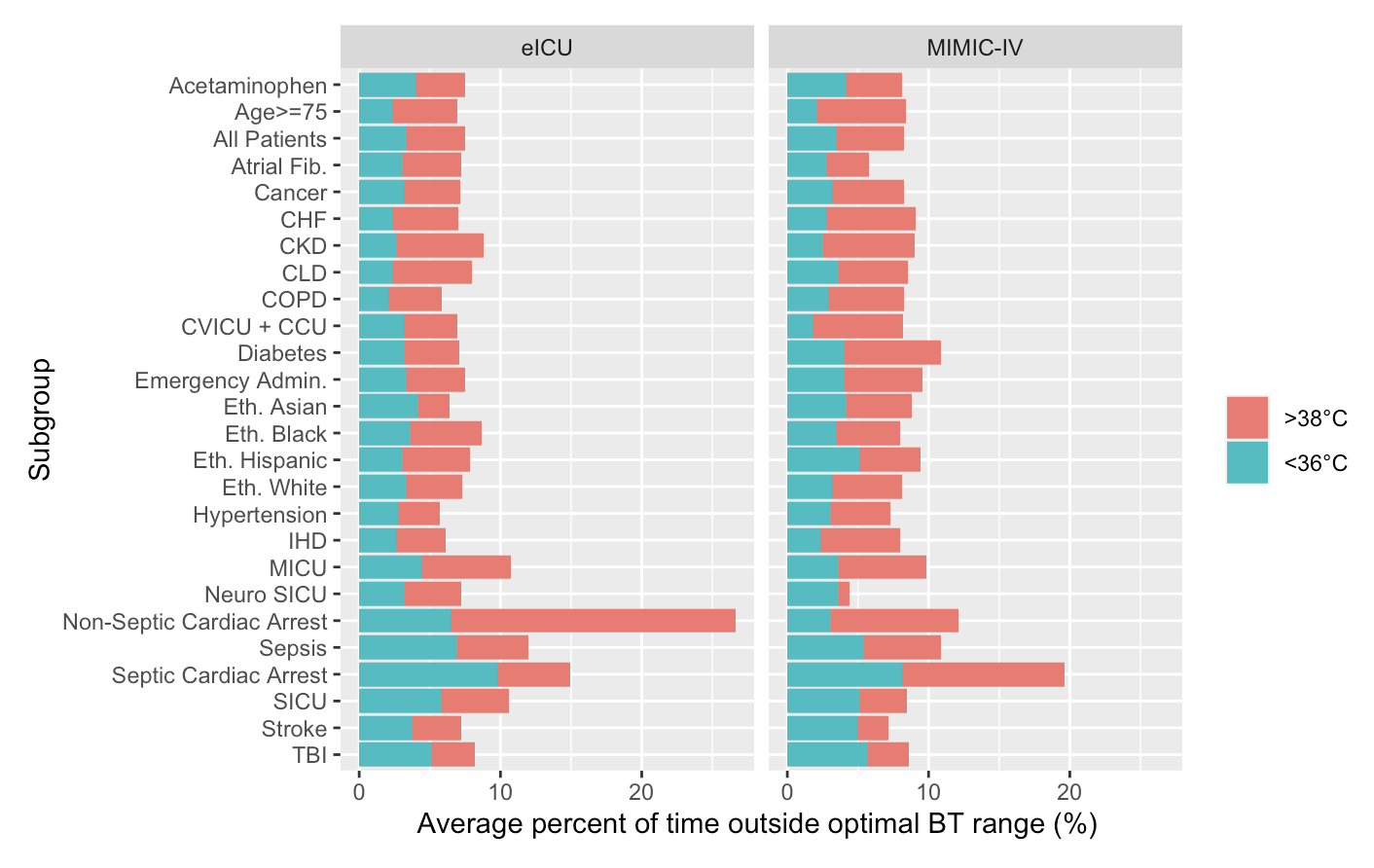

Supplement: Supplementary file 5 — Additional file 5: Figure S5. Average percent of time spent outside 36–36 °C per subgroup. [file 40001_2023_1616_MOESM5_ESM.jpg]
