# Supplementary material for: Association of body temperature and mortality in critically ill patients: an observational study using two large databases
Source: Eur J Med Res. 2024 Jan 6;29:33. doi: 10.1186/s40001-023-01616-3 (PMC10770998; doi:10.1186/s40001-023-01616-3)
Supplement: Supplementary file 6 — Additional file 6: Table S1. Results of (A) forward and (B) backwards stepwise regression using MIMIC-IV data with median BT as the main exposure and hospital mortality as the outcome. [file 40001_2023_1616_MOESM6_ESM.pdf]

e-Table 1: Results of **(A)** forward and **(B)** backwards stepwise regression using MIMIC-IV data for the with median BT as the main exposure and hospital mortality as the outcome.

A

Step	Exclusion	Covariate	AIC	BIC	adj.R-squared	P-val
Step 0	+	APS-III Score	20185	20202	0.2535	<0.0001
	+	SOFA Score	23670	23688	0.1245	<0.0001
	+	Vasopressor Use	25238	25255	0.0666	<0.0001
	+	Charlson Score	25606	25624	0.0529	<0.0001
	+	Temp Gap.	26075	26093	0.0356	<0.0001
	+	Ventilation Use	26204	26221	0.0308	<0.0001
	+	Age	26410	26427	0.0232	<0.0001
	+	Dialysis Use	26697	26714	0.0126	<0.0001
	+	Steroid Use	26872	26890	0.0061	<0.0001
	+	Median Temp.	26952	26969	0.0032	<0.0001
	+	Sex	27029	27046	0.0003	0.0021
		<none>	27036	27045	0	NA
Step 1: + APS-III Score	+	Charlson Score	19589	19615	0.2756	<0.0001
	+	Age	19741	19767	0.27	<0.0001
	+	Vasopressor Use	20016	20042	0.2598	<0.0001
	+	Steroid Use	20118	20144	0.256	<0.0001
	+	Ventilation Use	20132	20158	0.2555	<0.0001
	+	Median Temp.	20162	20188	0.2544	<0.0001
	+	Sex	20171	20197	0.254	<0.0001
	+	Temp Gap.	20174	20200	0.2539	0.0003
	+	Dialysis Use	20178	20204	0.2538	0.0032
	+	SOFA Score	20178	20204	0.2538	0.0031
		<none>	20185	20202	0.2535	NA
Step 2: + Charlson Score	+	Vasopressor Use	19383	19418	0.2833	<0.0001
	+	Age	19480	19514	0.2797	<0.0001
	+	Ventilation Use	19480	19514	0.2797	<0.0001
	+	Steroid Use	19537	19571	0.2776	<0.0001
	+	Temp Gap.	19538	19573	0.2775	<0.0001
	+	Dialysis Use	19572	19606	0.2763	<0.0001
	+	Sex	19572	19607	0.2763	<0.0001
	+	SOFA Score	19587	19622	0.2757	0.0523
	+	Median Temp.	19588	19623	0.2757	0.1263
		<none>	19589	19615	0.2756	NA
	-	APS-III Score	25606	25624	0.0529	<0.0001
Step 3: + Vasopressor Use	+	Age	19261	19304	0.2878	<0.0001
	+	Ventilation Use	19316	19359	0.2858	<0.0001
	+	Dialysis Use	19346	19390	0.2847	<0.0001
	+	Steroid Use	19347	19391	0.2846	<0.0001
	+	Temp Gap.	19354	19397	0.2844	<0.0001
	+	Sex	19365	19408	0.284	<0.0001
	+	SOFA Score	19374	19418	0.2836	0.0011
	+	Median Temp.	19383	19426	0.2833	0.1499
		<none>	19383	19418	0.2833	NA
	-	Charlson Score	20016	20042	0.2598	<0.0001
	-	APS-III Score	23870	23896	0.1172	<0.0001
Step 4: + Age	+	Ventilation Use	19187	19239	0.2906	<0.0001
	+	Steroid Use	19211	19263	0.2897	<0.0001
	+	Temp Gap.	19227	19279	0.2892	<0.0001
	+	Dialysis Use	19239	19291	0.2887	<0.0001
	+	Sex	19252	19304	0.2882	0.0011
	+	SOFA Score	19258	19310	0.288	0.0333
		<none>	19261	19304	0.2878	NA
	+	Median Temp.	19263	19315	0.2878	0.8847
	-	Vasopressor Use	19480	19514	0.2797	<0.0001
	-	Charlson Score	19536	19571	0.2776	<0.0001
	-	APS-III Score	23800	23835	0.1199	<0.0001

A cont.

Step	Exclusion	Covariate	AIC	BIC	adj.R-squared	P-val
Step 5: + Ventilation Use	+	Steroid Use	19137	19197	0.2926	<0.0001
	+	Dialysis Use	19165	19226	0.2915	<0.0001
	+	Temp Gap.	19171	19232	0.2913	<0.0001
	+	SOFA Score	19174	19235	0.2912	0.0001
	+	Sex	19177	19237	0.2911	0.0005
		<none>	19187	19239	0.2906	NA
	+	Median Temp.	19187	19248	0.2907	0.1600
	-	Age	19316	19359	0.2858	<0.0001
	-	Vasopressor Use	19361	19405	0.2841	<0.0001
	-	Charlson Score	19484	19528	0.2796	<0.0001
	-	APS-III Score	23320	23364	0.1377	<0.0001
Step 6: + Steroid Use	+	Dialysis Use	19114	19183	0.2935	<0.0001
	+	Temp Gap.	19119	19188	0.2933	<0.0001
	+	SOFA Score	19125	19195	0.293	0.0003
	+	Sex	19129	19198	0.2929	0.0020
		<none>	19137	19197	0.2926	NA
	+	Median Temp.	19138	19207	0.2926	0.3586
	-	Ventilation Use	19211	19263	0.2897	<0.0001
	-	Age	19280	19332	0.2872	<0.0001
	-	Vasopressor Use	19294	19346	0.2867	<0.0001
	-	Charlson Score	19414	19466	0.2822	<0.0001
	-	APS-III Score	23244	23296	0.1406	<0.0001
Step 7: + Dialysis Use	+	Temp Gap.	19095	19173	0.2942	<0.0001
	+	Sex	19106	19185	0.2938	0.0025
	+	SOFA Score	19109	19187	0.2937	0.0104
		<none>	19114	19183	0.2935	NA
	+	Median Temp.	19114	19192	0.2935	0.1695
	-	Steroid Use	19165	19226	0.2915	<0.0001
	-	Ventilation Use	19188	19249	0.2906	<0.0001
	-	Age	19240	19301	0.2887	<0.0001
	-	Vasopressor Use	19285	19345	0.2871	<0.0001
	-	Charlson Score	19405	19466	0.2826	<0.0001
	-	APS-III Score	23193	23254	0.1425	<0.0001
Step 8: + Temp Gap.	+	Sex	19088	19175	0.2945	0.0024
	+	SOFA Score	19090	19177	0.2945	0.0068
	+	Median Temp.	19094	19181	0.2943	0.0700
		<none>	19095	19173	0.2942	NA
	-	Dialysis Use	19119	19188	0.2933	<0.0001
	-	Steroid Use	19148	19218	0.2922	<0.0001
	-	Ventilation Use	19151	19221	0.2921	<0.0001
	-	Age	19225	19294	0.2893	<0.0001
	-	Vasopressor Use	19256	19325	0.2882	<0.0001
	-	Charlson Score	19399	19468	0.2829	<0.0001
	-	APS-III Score	22810	22880	0.1567	<0.0001
Step 9: + Sex	+	SOFA Score	19084	19180	0.2947	0.0150
	+	Median Temp.	19087	19182	0.2946	0.0713
		<none>	19088	19175	0.2945	NA
	-	Temp Gap.	19106	19185	0.2938	<0.0001
	-	Dialysis Use	19111	19190	0.2936	<0.0001
	-	Steroid Use	19138	19216	0.2926	<0.0001
	-	Ventilation Use	19145	19223	0.2924	<0.0001
	-	Age	19208	19286	0.29	<0.0001
	-	Vasopressor Use	19249	19327	0.2885	<0.0001
	-	Charlson Score	19398	19476	0.283	<0.0001
	-	APS-III Score	22794	22872	0.1573	<0.0001
Step 10: + SOFA Score	+	Median Temp.	19082	19187	0.2948	0.0574
		<none>	19084	19180	0.2947	NA
	-	Sex	19090	19177	0.2945	0.0052
	-	Dialysis Use	19101	19188	0.294	<0.0001
	-	Temp Gap.	19103	19190	0.294	<0.0001
	-	Steroid Use	19134	19220	0.2928	<0.0001
	-	Ventilation Use	19146	19233	0.2924	<0.0001
	-	Age	19201	19288	0.2903	<0.0001
	-	Vasopressor Use	19249	19335	0.2886	<0.0001
	-	Charlson Score	19399	19486	0.283	<0.0001
	-	APS-III Score	21876	21963	0.1914	<0.0001

B

	Exclusion	Covariate	AIC	BIC	adj.R-squared	P-val
**Step 0**		<none>	19082	19187	0.2948	
	−	Median Temp.	19084	19180	0.2947	0.0574
	−	SOFA Score	19087	19182	0.2946	0.0122
	−	Sex	19088	19184	0.2946	0.0053
	−	Dialysis Use	19101	19196	0.2941	<0.0001
	−	Temp Gap.	19103	19198	0.294	<0.0001
	−	Steroid Use.	19130	19225	0.293	<0.0001
	−	Ventilation Use	19147	19242	0.2924	<0.0001
	−	Age	19192	19288	0.2907	<0.0001
	−	Vasopressor Use	19246	19342	0.2887	<0.0001
	−	Charlson Score	19396	19491	0.2832	<0.0001
	−	APS-III Score	21860	21955	0.192	<0.0001
