# Supplementary material for: Association of body temperature and mortality in critically ill patients: an observational study using two large databases
Source: Eur J Med Res. 2024 Jan 6;29:33. doi: 10.1186/s40001-023-01616-3 (PMC10770998; doi:10.1186/s40001-023-01616-3)
Supplement: Supplementary file 7 — Additional file 7: Table S2. Crude odds ratio of mortality at different temperature ranges. [file 40001_2023_1616_MOESM7_ESM.docx]

**e-Table 2:** Crude odds ratio of mortality at different temperature ranges.

|  | **MIMIC-IV** | | **eICU** | |
| --- | --- | --- | --- | --- |
| Temperature | Hospital Mortality | ICU Mortality | Hospital Mortality | ICU Mortality |
| <36°C | 1.22 (1.2-1.24) | 1.26 (1.24-1.29) | 1.35 (1.34-1.37) | 1.4 (1.38-1.41) |
| 36°C - 38°C | 0.83 (0.82-0.84) | 0.79 (0.78-0.81) | 0.77 (0.76-0.78) | 0.74 (0.73-0.75) |
| >38°C | 1.16 (1.14-1.18) | 1.2 (1.18-1.23) | 1.18 (1.17-1.2) | 1.21 (1.19-1.23) |
